# Understanding suicidal ideation disparity between sexual minority and heterosexual Chinese young men: a multiple mediation model of social support sources, self-esteem, and depressive symptoms

**DOI:** 10.3389/fpsyt.2024.1265722

**Published:** 2024-03-15

**Authors:** Yiting Huang, Jiayu Liu, Gang Huang, Dejia Zhu, Yunfei Zhou, Jingchu Hu

**Affiliations:** ^1^ Department of Anxiety Disorders, Shenzhen Mental Health Center, Shenzhen Kangning Hospital, Shenzhen, Guangdong, China; ^2^ Graduate School of Arts & Science, New York University, New York, NY, United States; ^3^ College of Literature, Science, and the Arts, University of Michigan, Ann Arbor, MI, United States

**Keywords:** suicidal ideation, Chinese sexual minority, young men, social support sources, self-esteem, depressive symptoms

## Abstract

**Objectives:**

Although sexual minorities have reported higher levels of suicidal ideation than heterosexuals across cultures, the role of various psychosocial factors underlying this disparity among young men has been understudied, particularly in China. This study examined the multiple mediating effects of psychosocial factors between sexual orientation and suicidal ideation in Chinese sexual minority and heterosexual young men.

**Methods:**

302 Chinese cisgender men who identified as gay or bisexual, and 250 cisgender heterosexual men (n=552, aged 18-39 years) completed an online questionnaire measuring perceived social support, self-esteem, depressive symptoms, and suicidal ideation.

**Results:**

Young sexual minority men reported significantly higher suicidal ideation and lower social support than their heterosexual peers. Structural equation modelling revealed two multiple indirect pathways. One pathway indicated that sexual orientation was indirectly related to suicidal ideation via family support and depressive symptoms. Another pathway indicated that sexual orientation was indirectly related to suicidal ideation via support from friends, self-esteem, and depressive symptoms.

**Conclusions:**

This study is among the first to examine the potentially cascading relationships between sexual orientation and psychosocial factors with suicidal ideation in a Chinese sample of young men. The findings highlight several promising psychosocial targets (i.e., improving family/friend support and increasing self-esteem) for suicide interventions among sexual minority males in China.

## Introduction

The mental health challenges faced by individuals in sexual minority groups are becoming an increasingly significant public health issue (1–4). Results from the meta-analysis demonstrated a greater prevalence of depression and suicidality in sexual minority individuals than in heterosexuals in both Western countries and China (5–9). In addition, adverse mental health outcomes are most common among young adults, which could include suicide, the second leading cause of youth death globally (10). Youths with Lesbian, Gay, Bisexual & Transgender (LGBT) identities suffer more psychological distress (11–13). Meyer proposed a minority stress model as the theoretical framework to explain these differences caused by their disadvantaged sexual orientation status (14–16). Hatzenbuehler also proposed a psychological mediation framework and postulated that the mediation effects of social and cognitive processes for sexual minority individuals confer the risk for psychopathology (17). However, most of their theories are based on Western samples, and the associations between sexual orientation and negative mental health outcomes and the potential mechanisms underlying this association in young men are understudied in China.

Moreover, while there is good evidence of poor mental health outcomes and a higher rate of suicidality among sexual minority people, few studies investigate the social determinants of this disparity in China. Thus, the current study has three parts. First, the study will investigate the difference in suicidal ideation between sexual minority and heterosexual young men in China. Second, the study aims to examine the psychosocial factors through which sexual orientation leads to psychopathology (depressive symptoms and suicidal ideation) among Chinese sexual minority young men and heterosexual young men. Finally, the study will explore the role of depressive symptoms in the association between sexual orientation and suicidal ideation.

### The mediating effect of social support

In Meyer’s model and social network theory, social support is an essential coping resource and an integral part of the minority stress process (15, 18). Individuals who perceived support from family members, friends, or others tended to be mentally healthier than those who lacked social support (19–22). However, marginalized social groups like sexual minority people are exposed to more stress and receive less social support than their heterosexual counterparts (23, 24). Less social support is associated with higher depressive symptoms and suicidal ideation among sexual minority people, as reported in recent cross-sectional studies (22, 25–30). The psychological mediation framework conceptualized social support as a mediator of the stress-psychopathology relationship (17). Although a cross-sectional study discovered a full mediation effect of social support from family in the association between sexual orientation and depressive symptoms (31), there has been less investigation of different sources of support contributing to possible consequences of sexual orientation.

Up to recently, only a few studies examined the impacts of different sources of social support on mental health in LGBT youth. For example, the study by Needham and Austin found that parental support partially mediated the relationship between gay identity and suicidal ideation in gay and heterosexual young adults (32). Additionally, another study indicated that the total score of perceived social support fully mediated sexual orientation victimization and depression among LGB youths (33). Given the essential role of the family in Chinese Confucian culture, it is probable that support from family, friends, and others may have a different impact on the association. The present study wants to determine whether different dimensions of perceived social support account for different mediation effects in the Chinese population on the relationship between sexual orientation and depressive symptoms, which in turn influence suicidal ideation.

### The mediating effect of self-esteem

The psychological mediation framework also conceptualized self-esteem as a related cognitive risk factor in developing psychological distress, including suicidality (17). Recent evidence suggests that self-esteem is another critical psychosocial variable indirectly affecting the well-being and psychopathology of the sexual minority population (16, 29, 34, 35). Indeed, low self-esteem is likely to be caused by not receiving enough social support, which in turn confers the risk of psychological problems. Relatedly, former studies demonstrated that insufficient social support was associated with negative self-esteem in the sexual minority group (22, 29). Researchers also identified that self-esteem mediated the relationship between perceived social support and depressive symptoms among human immunodeficiency virus (HIV)-positive men who had sex with men in China (36).

Further, a study on sexual minority youth in the United Kingdom showed that depressive symptoms worked as a mediator between self-esteem and suicidal ideation (16). Another study of Chinese LGB individuals also demonstrated that self-esteem mediated the influence of friend support on psychological distress (37). However, to date, no studies have examined the mediating role of self-esteem and different sources of social support in the association between sexual orientation, depression, and suicidal ideation in the Chinese population.

### Present investigation

**Figure 1:**
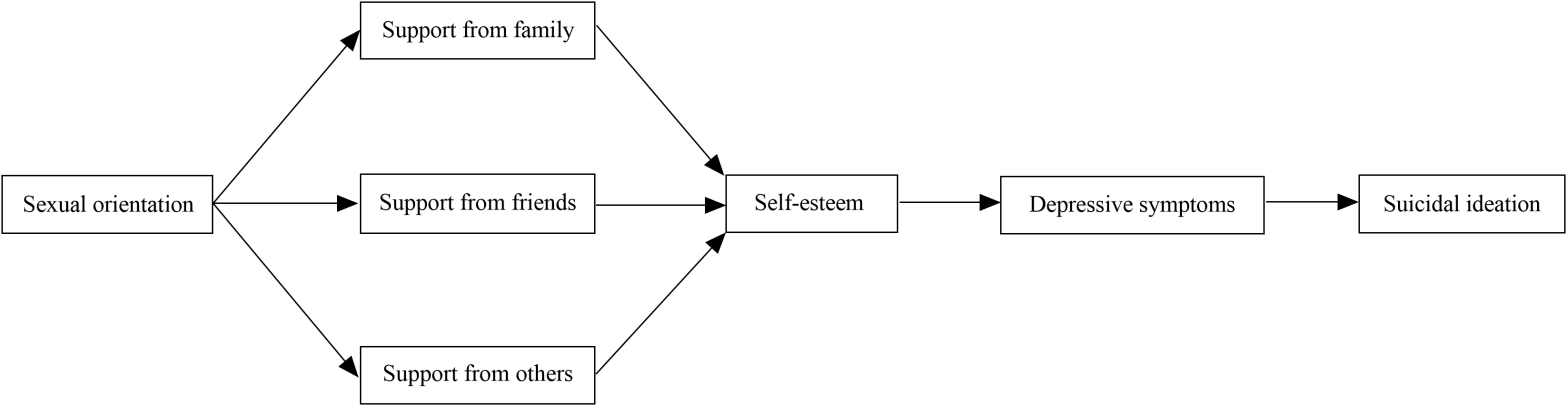
Conceptual Model between sexual orientation and suicidal ideation.

In the current study, we intend to extend previous studies by examining the mediating roles of different sources of perceived social support and self-esteem in the association between sexual orientation and depressive symptoms, consequently affecting suicidal ideation among Chinese young men. None of the research has studied these associations, especially in the Chinese sexual minority population. Since same-sex marriage has not been legalized in China, some Chinese people still discriminate against the sexual minority population (38, 39). Chinese sexual minorities may face more minority stress than the sexual minority group in Western countries. To better understand the psychological well-being of the sexual minority population, the present study proposed a conceptual model to explore the link between sexual orientation and suicidal ideation (shown in **Figure 1**). Based on prior research and previous findings, this study had three following hypotheses:

(1) Compared to heterosexual men, sexual minority men would perceive less social support, lower self-esteem, more depressive symptoms, and more suicidal ideation.
(2) Sexual orientation (code 1 as heterosexual status and 2 as sexual minority status in the final data analysis) will be positively associated with suicidal ideation and depressive symptoms but negatively correlated with social support and self-esteem.
(3) Perceived social support, self-esteem, and depressive symptoms sequentially mediate the association between sexual orientation and suicidal ideation. Further, different sources of perceived social support may serve different mediating roles in the conceptual model.

## Methods

### Participants

794 Chinese participants completed the survey, and 552 valid questionnaires were included in the data analysis. Given our focus on sexual minority and heterosexual men, the exclusion criteria (N = 242) were as follows: a. insufficient number of female participants (N = 57); b. missing data on age (N = 69); c. mismatch between biological sex and self-identified gender (N = 93); d. self-reported as unsure of their sexual orientation (N = 2); e. age under 18 or over 40 (N = 21). The final sample consisted of 250 cisgender heterosexual men (who identified themselves as heterosexual) with an age mean, median of 23.89, 23 (*SD* = 3.98), and 302 cisgender sexual minority men (50 men identified themselves as bisexual, 252 men identified themselves as gay) with an age mean, median of 23.99, 23 (*SD* = 3.68). Demographic information is presented in **Table 1**. The dataset can be found in **Supplementary Material**.

**Table 1:** Descriptive statistics for study variables of the sample (n = 552).

	Heterosexual (n = 250)	Sexual minority (n = 302)	*χ^2^/t*	*P-value*
Demographics				
Age *mean (SD*)	23.89 (3.98)	23.99 (3.68)	- 0.33	0.74
Whether only-one child n (%)			1.70	0.19
Only child	131 (52.4)	175 (57.9)		
Has siblings	119 (47.6)	127 (42.1)		
Heterosexual marital status & intention n (%) ^a^			273.67	< 0.001
Married	43 (17.2)	3 (1)		
Single without marriage intention	6 (2.4)	140 (46.4)		
Single with marriage intention	137 (54.8)	20 (6.6)		
Single and not sure about marriage	63 (25.2)	135 (44.7)		
Divorced will not marry again	0 (0)	2 (0.7)		
Divorced will marry again	1 (0.4)	0 (0)		
Widowed	0 (0)	2 (0.7)		
Education level n (%)			8.30	0.08
Primary school or lower	1 (0.4)	0 (0)		
Junior high school	2 (0.8)	2 (0.7)		
Senior high school	16 (6.4)	24 (7.9)		
College	211 (84.4)	231 (76.5)		
Post-graduate or higher	20 (8)	45 (14.9)		
Occupation n (%)			5.60	0.59
Student	118 (47.2)	137 (45.4)		
Unemployed	7 (2.8)	12 (4)		
Unstable employee	6 (2.4)	8 (2.6)		
Regular employee	65 (26)	90 (29.8)		
Service industry	10 (4)	18 (6)		
Teacher or government personnel	18 (7.2)	18 (6)		
Medical staff or army	5 (2)	4 (1.3)		
Other	21 (8.4)	15 (5)		
Income (¥/month) n (%)			2.47	0.78
< 2,000	113 (45.2)	118 (39.1)		
2,000 – 4,000	62 (24.8)	79 (26.2)		
4,001 – 6,000	30 (12)	44 (14.6)		
6,001 – 8,000	17 (6.8)	24 (7.9)		
8,001 – 10,000	11 (4.4)	16 (5.3)		
>10,000	17 (6.8)	21 (7)		
Main variables mean (SD)				
Self-esteem	31.32 (4.75)	31.67 (5.02)	- 0.83	0.41
Social support	61.85 (11.67)	56.09 (13.40)	5.40	< 0.001
Social support from family ^b^	21.13 (5)	17 (5.53)	9.21	< 0.001
Social support from friends ^b^	20.96 (4.22)	19.98 (5.07)	2.46	0.01
Social support from significant others ^b^	19.76 (4.58)	19.11 (5.04)	1.61	0.11
Depressive symptoms	17.90 (11.09)	19.60 (10.41)	- 1.84	0.07
Suicidal ideation	1.21 (0.45)	1.40 (0.58)	- 4.32	< 0.001

^a^The marital status in this article refers to heterosexual marriage, as same-sex marriage is not currently legally recognized in China; ^b^ Dimension of Multidimensional Scale of Perceived Social Support (MSPSS).

### Procedures

The Chinese sexual minority participants were recruited from the LGBT social groups on the following Chinese online platforms: QQ application and douban.com. To maximize the comparability of participants, we recruited heterosexual participants on the same platforms (QQ application and douban.com). These participants were blind as to the comparison but knew the general purpose of the study. Chi-squared analyses and independent *t*-tests demonstrated no difference between the groups in age, whether only child, education level, occupation, or income. These groups did differ significantly in marital status.

The present study was a cross-sectional online survey approved by the Institutional Review Board of Kangning Hospital. All participants needed to fill out the informed consent form before the survey and were informed of the right to withdraw from the survey at any time. After the survey, all participants were compensated with a small reward by Alipay [10 Chinese yuan renminbi (CNY), approximately 1.5 U.S. dollars (USD)].

### Measures

Social support. The Chinese version of the Multidimensional Scale of Perceived Social Support (MSPSS) was used to examine participants’ perceived social support (40). The MSPSS is a 12-item self-reported scale with three dimensions: family, friend, and others. This is a seven-point Likert scale from 1 = very strongly disagree to 7 = very strongly agree. The average score of the three dimensions presents the participants’ MSPSS score, and a higher MSPSS score indicates a higher perceived social support in family, friends, or others. For the present study, Cronbach’s α coefficient for the MSPSS was 0.92, and for the three subscales: support from family was 0.87, support from friends was 0.89, and support from others was 0.84.

Self-esteem. The Chinese version of the Self-esteem Scale (SES) was used to evaluate participants’ self-esteem levels (41). The questionnaire includes ten items measured on a four-point Likert-type scale, ranging from 1 = strongly disagree to 4 = strongly agree. The higher average of the total score suggests a higher level of self-esteem. The Cronbach’s α of this scale was 0.88.

Depressive symptoms. The Chinese version of the Center for Epidemiologic Studies-Depression Scale (CES-D) was used to measure participants’ depressive symptoms during the last week (42). The CES-D scale consists of 20 items, rating from 0 = rarely or none of the time to 3 = most or all the time. The total score is 60, and a higher score indicates more severe depressive symptoms. The Cronbach’s α coefficient for this measure was 0.90.

Sexual orientation. The participants’ sexual orientation was assessed by the question: “What is your sexual orientation?”. The alternative answers were heterosexual, gay and lesbian, bisexual, transgender, and unsure. Participants who identified themselves as gay, lesbian, or bisexual were combined and entered as the sexual minority group. Participants who identified themselves as heterosexual were analyzed as the heterosexual group (1= heterosexual, 2 = sexual minority). Two participants who were not sure about their sexual orientation were excluded from the current study. None of the participants identified as transgender.

Suicidal ideation. Suicidal ideation was assessed by the question: “Do you have any suicidal thoughts during the past 12 months?”. Participants were asked to rate from three levels: 0 = Never, 1 = Sometimes, and 2 = Often.

### Statistical analysis

First, IBM SPSS statistical version 26.0 (IBM Corp) was used to describe demographic information, main variables in sexual minority and heterosexual groups, and main variables inter-correlations. We used independent sample t-tests for continuous variables, Chi-square tests for categorical variables, and Point-biserial for correlations. Second, Mplus 8.3 was used to evaluate the conceptual multiple mediation model by conducting structural equation modeling. The latent variables of support from family, support from friends, self-esteem, and depressive symptoms were indicated by items. Demographics (age, marital status, whether only-one child, education level, occupation, and income) were entered as control variables, and the independent variable sexual orientation was coded as 1= heterosexual and 2 = sexual minority in model testing. Bootstrapping was set at 5000 samples and evaluated the model fit with the following indices: *χ^2^/df* ratio (lower than 3), Root Mean Square Error of Approximation (RMSEA; lower than 0.08), Tucker–Lewis Index (TLI; greater than 0.90), and Comparative Fit Index (CFI; greater than 0.09) (43).

## Results

### Descriptive and correlation results

The demographic information and the difference in main variables were summarized in **Table 1**. The results indicated a significant difference in marital status (*χ^2 =^* 273.67, *p* < 0.001) between heterosexual and sexual minority groups. Similar to the previous study, sexual minority participants showed less marriage intention than heterosexual participants in the current study (35). The differences in other demographics were not significant between groups (*ps* > 0.05). For the key variables, participants in the sexual minority group reported a significantly lower level of social support (*t* = 5.40, *p* < 0.001), social support from family (*t* = 9.21, *p* < 0.001), social support from friends (*t* = 2.46, *p* = 0.01) than heterosexual; and more suicidal ideation (*t* = - 4.32, *p* < 0.001) than heterosexual.

**Table 2** shows the intercorrelations of the main variables in all participants. Sexual orientation was negatively correlated with social support (*r* = - 0.22, *p* < 0.001), social support from family(*r* = - 0.38, *p* < 0.001), and social support from friends (*r* = - 0.09, *p* = 0.045); was positively correlated with suicidal ideation (*r* = 0.18, *p* < 0.001). Self-esteem was positively correlated with support from family (*r* = 0.28, *p* < 0.001) and support from friends (*r* = 0.34, *p* < 0.001). Depressive symptoms were positively correlated with suicidal ideation (*r* = 0.32, *p* < 0.001); were negatively correlated with self-esteem (*r* = - 0.53, *p* < 0.001).

**Table 2:** Intercorrelations among the study variables.

	1	2	3	4	5	6	7	8
1. Sexual orientation	–							
2. Social support	-0.22^***^	–						
3. Social support from family	-0.38^***^	0.81^***^	–					
4. Social support from friends	-0.09^*^	0.85^***^	0.50^***^	–				
5. Social support from significant others	-0.05	0.85^***^	0.47^***^	0.75^***^	–			
6. Self-esteem	0.04	0.36^***^	0.28^***^	0.34^***^	0.30^***^	–		
7. Depressive symptoms	0.10^*^	-0.38^***^	-0.35^***^	-0.35^***^	-0.30^***^	-0.53^***^	–	
8. Suicidal ideation	0.18^***^	-0.24^***^	-0.24^***^	-0.17^***^	-0.17^***^	-0.29^***^	0.32^***^	–

^*^p <.05; ^***^p <.001.

### Model testing

**Figure 2:**
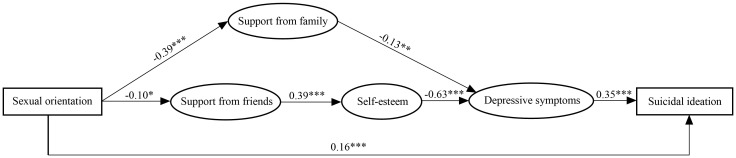
Path diagram for the relationship between sexual orientation and suicidal ideation. Coefficients are standardized and adjusted for age, education, occupation, marital status, income, and whether only-one child. Note. Support from family and Support from friends are the two dimensions of the Multidimensional Scale of Perceived Social Support (MSPSS). Self-esteem presents the Chinese version of the Self-esteem Scale (SES); Depressive symptoms present the Chinese version of the Center for Epidemiologic Studies-Depression Scale (CES-D). Latent variables (enclosed in oval-shaped frames) are measured by the observable variables (scale items). Note. *p < .05; **p < .01; ***p < .001.

We examined the conceptual multiple multiple mediation model mentioned in the introduction, investigating three possible indirect pathways in the association of sexual orientation and suicidal ideation. The results of the conceptual model suggested that the pathway that included support from others was insignificant (*B* = 0.002, *p* = 0.31). Therefore, this pathway was deleted from the final model. We confirmed the final structural model (**Figure 2**), including two indirect pathways and a direct pathway, and all indices indicate good model fit: χ^2^/*df* = 2.06, RMSEA = 0.04, TLI = 0.90, CFI = 0.91. The parameter estimate of the final model is presented in **Table 3**. The direct effect of sexual orientation and suicidal ideation was 0.18 (95% CI = 0.11, 0.26), and the total indirect effect was 0.03 (95% CI = 0.01, 0.05). The model revealed that sexual orientation (identifying as 1 = heterosexual or 2 = sexual minority) was negatively related to support from family (*B* = - 0.39, *p* < 0.001) and support from friends (*B* = - 0.10, *p* = 0.03). Support from friends (*B* = 0.39, *p* < 0.001) was positively related to self-esteem. Both self-esteem (*B* = - 0.63, *p* < 0.001) and support from family (*B* = - 0.13, *p* = 0.004) were negatively related to depressive symptoms. More depressive symptoms predicted more suicidal ideation (*B* = 0.35, *p* < 0.001).

**Table 3:** Standardized parameter estimate for the final structural model.

	B(*SE*)	95% CI
Total effect		
Sexual orientation → Suicidal ideation	0.18(0.04)^***^	[0.11, 0.26]
**Total indirect effect**		
Sexual orientation → Suicidal ideation	0.03(0.01)^**^	[0.01, 0.05]
Direct path		
Sexual orientation *→* Suicidal ideation	0.16(0.04)^***^	[0.08, 0.24]
Indirect path		
Sexual orientation → Support from family → Depressive symptoms → Suicidal ideation	0.02(0.01)^**^	[0.01, 0.03]
Sexual orientation → Support from friends → Self-esteem→ Depressive symptoms → Suicidal ideation	0.01(0.004)^*^	[0.001, 0.02]

Demographic variables (age, heterosexual marital status, whether only-one child, education level, occupation, and income) are controlled as covariates the mediation analysis. ^*^p <.05; ^**^p <.01; ^***^p <.001.

## Discussion

Using a sample of sexual minority and heterosexual young men in mainland China, this study was one of the first to examine the multiple mediating roles of different sources of social support, self-esteem, and depressive symptoms in the association between sexual orientation and suicidal ideation. We found two mediating pathways for the association. One pathway indicated that Chinese sexual minority men endorsed less family support in their social networks, which mediated the relationship between sexual orientation and depressive symptoms, resulting in more suicidal ideation. Another pathway indicated that lower friend support in Chinese sexual minority young men led to lower self-esteem than in heterosexuals, which in turn predicted depressive symptoms and accounted for more suicidal ideation. These results created a path model linking sexual orientation to suicidal ideation through a series of psychosocial factors for Chinese young men.

Consistent with previous studies, our findings showed that sexual minority young men reported significantly less social support, marginally more depressive symptoms, and more suicidal ideation compared to heterosexual men (7–9, 44). The mediation analysis conducted in this study uncovered significant chain mediating effects of social support, self-esteem, and depressive symptoms on the association between sexual orientation and suicidal ideation. It is important to note that only support from family and friends contributed to the mediation effect in the association but not support from others. Unlike previous western studies unveiling the mediation effect of the total score of social support or only the score of family support, our results showed that family support and friends’ support are prominent protective factors in Chinese sexual minority young men (32, 33). Individuals with lower family support and friends’ support may face more challenges and become more vulnerable to depressive symptoms and suicidal ideation (35, 45, 46). An explanation that can account for these results may be that in Asian culture, sexual minority youth were more likely to rely more on family and friends’ support rather than support from others.

Notably, self-esteem only mediated support from friends but not support from family. Given that peers play an important part in young adult life, peer support can increase people’s self-esteem and sense of control (47). Chinese sexual minority young men with a lower level of support from friends experienced lower self-esteem, which was associated with more depressive symptoms and more suicidal ideation. Altogether, young sexual minority men in China lacking family support and peer support may be particularly vulnerable to self-esteem and at risk for depressive symptoms and suicidal ideation.

### Limitations

Several limitations to the current study should be acknowledged. First, the study was cross-sectional, which could not make causal inferences. Longitudinal methodologies are needed to verify the causality. In addition, we only studied homosexual and bisexual cisgender men as the sexual minority group. It is unknown whether this conclusion can be generalized to lesbian groups, asexuals, or transgender. Finally, due to the difficulty of collecting data from special social groups, our sample size may not be large enough to represent sexual minority individuals in China.

### Implications

Nevertheless, the results of the current study may have important implications. First, these findings contribute to the literature on suicidality among Chinese sexual minority young men and may promote the development of interventions for high-risk individuals. Second, interventions to prevent adverse mental health outcomes by enhancing family and friend support may yield better outcomes. Third, self-esteem and perceived family and friend support may be valuable measures for psychological counseling of sexual minority youth at risk for depressive symptoms and suicidal ideation. Finally, LGBTQ+ movements, such as independent pride events for sexual minority groups in China, are needed to promote self-esteem.

## Conclusions

These results showed that different sources of social support explained different pathways in the development of suicidal ideation among Chinese sexual minority men. First, family support mediated the relationship between sexual orientation, depressive symptoms, and suicidal ideation. In contrast, friend support mediated the relationship between sexual orientation and self-esteem, which in turn conferred risk for depressive symptoms and suicidal ideation. They suggest that interventions to reduce suicidal ideation and depressive symptoms among young sexual minority men should focus on enhancing social support from family and friends and promoting their self-esteem in Chinese society.

## Data availability statement

The original contributions presented in the study are included in the article/**Supplementary Material**, further inquiries can be directed to the corresponding author/s.

## Ethics statement

The studies involving humans were approved by Institutional Review Board of Kangning Hospital. The studies were conducted in accordance with the local legislation and institutional requirements. The participants provided their written informed consent to participate in this study.

## Author contributions

YH: Formal analysis, Methodology, Writing – original draft. JL: Formal analysis, Methodology, Writing – review & editing. GH: Data curation, Investigation, Writing – review & editing. DZ: Validation, Writing – review & editing. YZ: Conceptualization, Funding acquisition, Supervision, Writing – review & editing. JH: Conceptualization, Funding acquisition, Methodology, Supervision, Writing – review & editing.
